# Supplementary material for: Sarcoconvolutums F and G: Polyoxygenated Cembrane-Type Diterpenoids from Sarcophyton convolutum, a Red Sea Soft Coral
Source: Molecules. 2022 Sep 8;27(18):5835. doi: 10.3390/molecules27185835 (PMC9500913; doi:10.3390/molecules27185835)
Supplement: Supplementary file 1 [file molecules-27-05835-s001.zip › molecules-1855511-supplementary.pdf]

Article

Sarcoconvolutums F and G: Polyoxygenated Cembrane-Type Diterpenoids from *Sarcophyton convolutum*, a Red Sea Soft Coral

Tarik A. Mohamed [1], Abdelsamed I. Elshamy [2], Mohamed H. Abd El-Razek [2], Asmaa M. Abdel-Tawab [3], Sherin K. Ali [1], Mohamed Aboelmagd [4], Midori Suenaga [5], Paul W. Pare [6], Akemi Umeyama [5] and Mohamed-Elamir F. Hegazy [1,*]

[1] Chemistry of Medicinal Plants Department, National Research Centre, 33 El-Bohouth St., Dokki, Giza 12622, Egypt
[2] Department of Natural Compounds Chemistry, National Research Centre, 33 El-Bohouth St., Dokki, Giza 12622, Egypt
[3] Marine Biotechnology and Natural Products Laboratory, National Institute of Oceanography and Fisheries, Cairo 11516, Egypt
[4] Pharmacognosy Department, National Research Centre, 33 El-Bohouth St., Dokki, Giza 12622, Egypt
[5] Faculty of Pharmaceutical Sciences, Tokushima Bunri University, Yamashiro-cho, Tokushima 770-8514, Japan
[6] Department of Chemistry & Biochemistry, Texas Tech University, Lubbock, TX 79409, USA
* Correspondence: mohegazy@uni-mainz.de; Tel.: +2-033-371-635

Supporting data	Page
Figure S1:^{1}H NMR ($CDCl_3$, 500 MHz) of **1** ……………………………..	3
Figure S2:^{13}C NMR ($CDCl_3$, 125 MHz) of **1** …………………………….	4
Figure S3:DEPT of **1** ……………………………………………………...	5
Figure S4:HSQC of **1** ……………………………………………………..	6
Figure S5:HMBC of **1**…………………………………………………….	7
Figure S6: ^{1}H-^{1}H COSY of **1**…………………………………………….	8
Figure S7:NOESY**1**……………………………………………………...	9
Figure S8: HRCI/MS **1**…………………………………………………...	10
Figure S9:^{1}H NMR ($CDCl_3$, 500 MHz) of **2** ……………………………..	13
Figure S10:^{13}C NMR ($CDCl_3$, 125 MHz) of **2** …………………………….	14
Figure S11:DEPT of **2** …………………………………………………...	16
Figure S12:HSQC of **2** …………………………………………………..	17
Figure S13:HMBC of **2**………………………………………………….	18
Figure S14: ^{1}H-^{1}H COSY of **2**……………………………………………	19
Figure S15:NOESY **2**……………………………………………………...	20
Figure S16: CI/MS **2**……………………………………………………...	21
Figure S17: HRCI/MS **2**…………………………………………………...	19
Figure S18:^{1}H NMR ($CDCl_3$, 500 MHz) of **3** ……………………………..	20
Figure S19:^{13}C NMR ($CDCl_3$, 125 MHz) of **3** …………………………….	21
Figure S20:^{1}H NMR ($CDCl_3$, 500 MHz) of **4**……………………………..	22
Figure S21:^{13}C NMR ($CDCl_3$, 125 MHz) of **4** …………………………….	23
Figure S22:^{1}H NMR ($CDCl_3$, 500 MHz) of **5** ……………………………..	24
Figure S23:^{13}C NMR ($CDCl_3$, 125 MHz) of **5** …………………………….	25
Figure S24:^{1}H NMR ($CDCl_3$, 500 MHz) of **6** ……………………………..	26
Figure S25:^{13}C NMR ($CDCl_3$, 125 MHz) of **6** …………………………….	27
Figure S26:^{1}H NMR ($CDCl_3$, 500 MHz) of **7** ……………………………..	28
Figure S27:^{13}C NMR ($CDCl_3$, 125 MHz) of **7** …………………………….	29

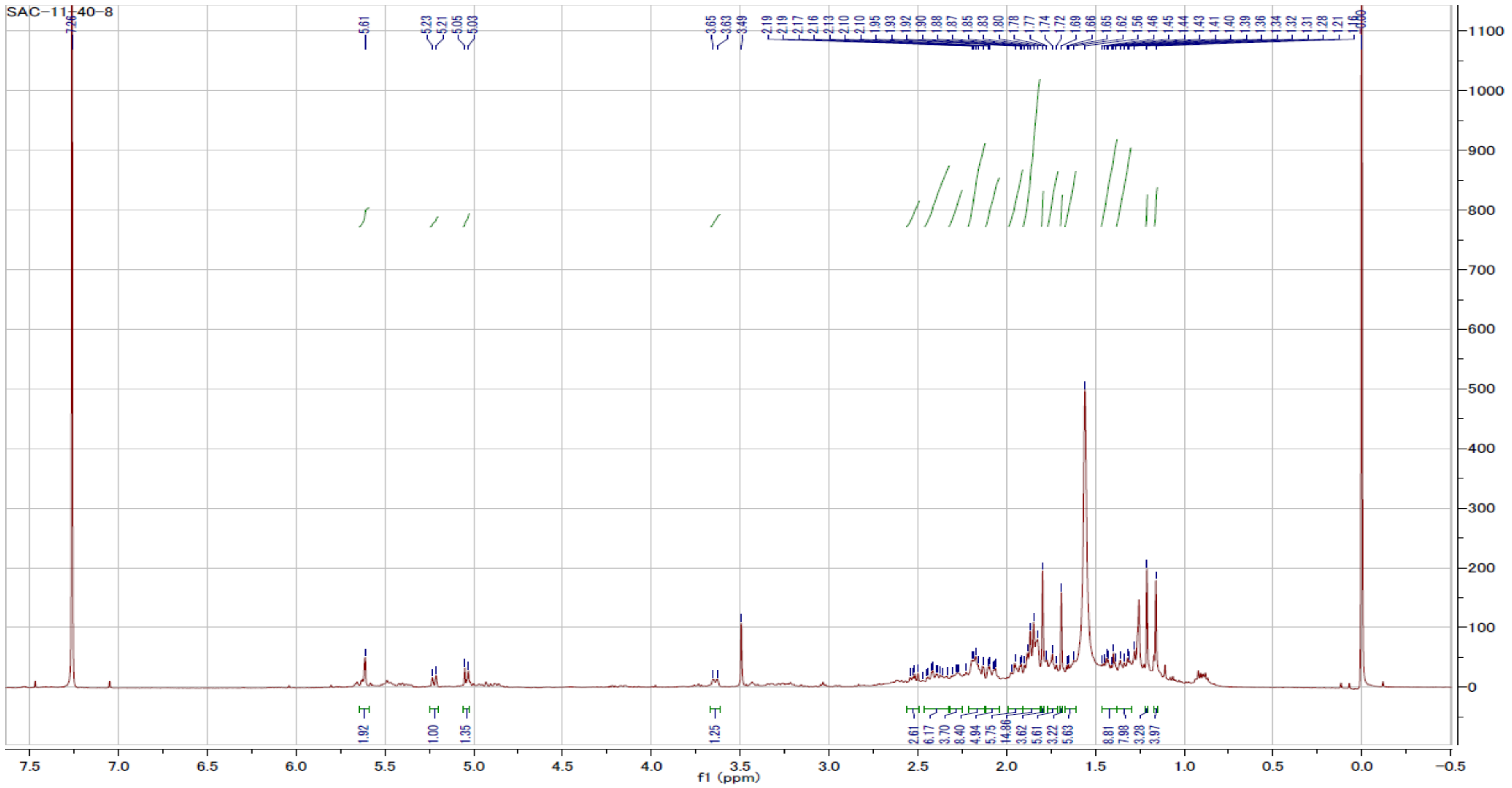

Figure S1. H NMR (CDCl3, 500 MHz) of **1.**

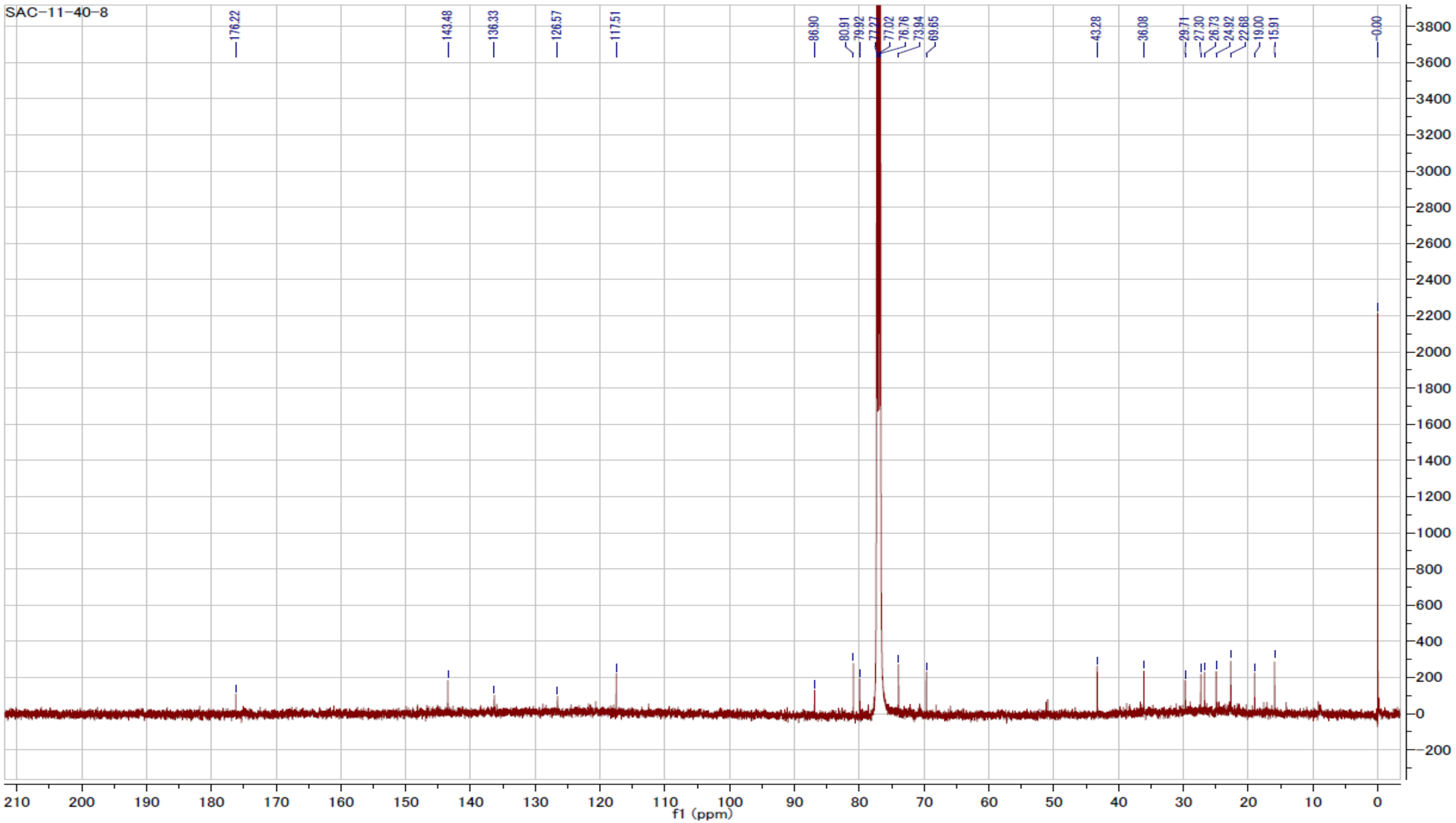

Figure S2. C NMR (CDCl3, 125 MHz) of **1.**

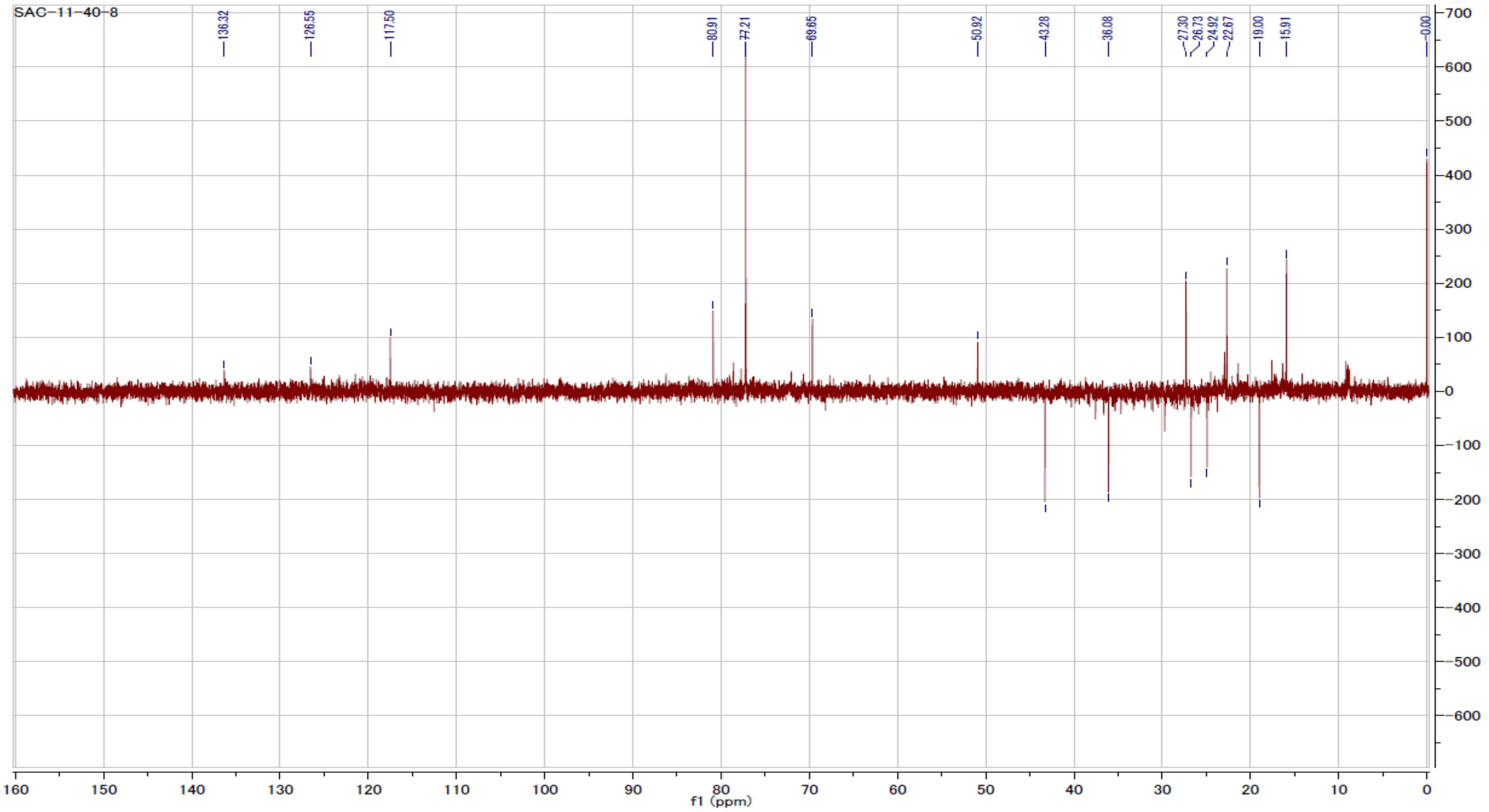

Figure S3. DEPT of **1.**

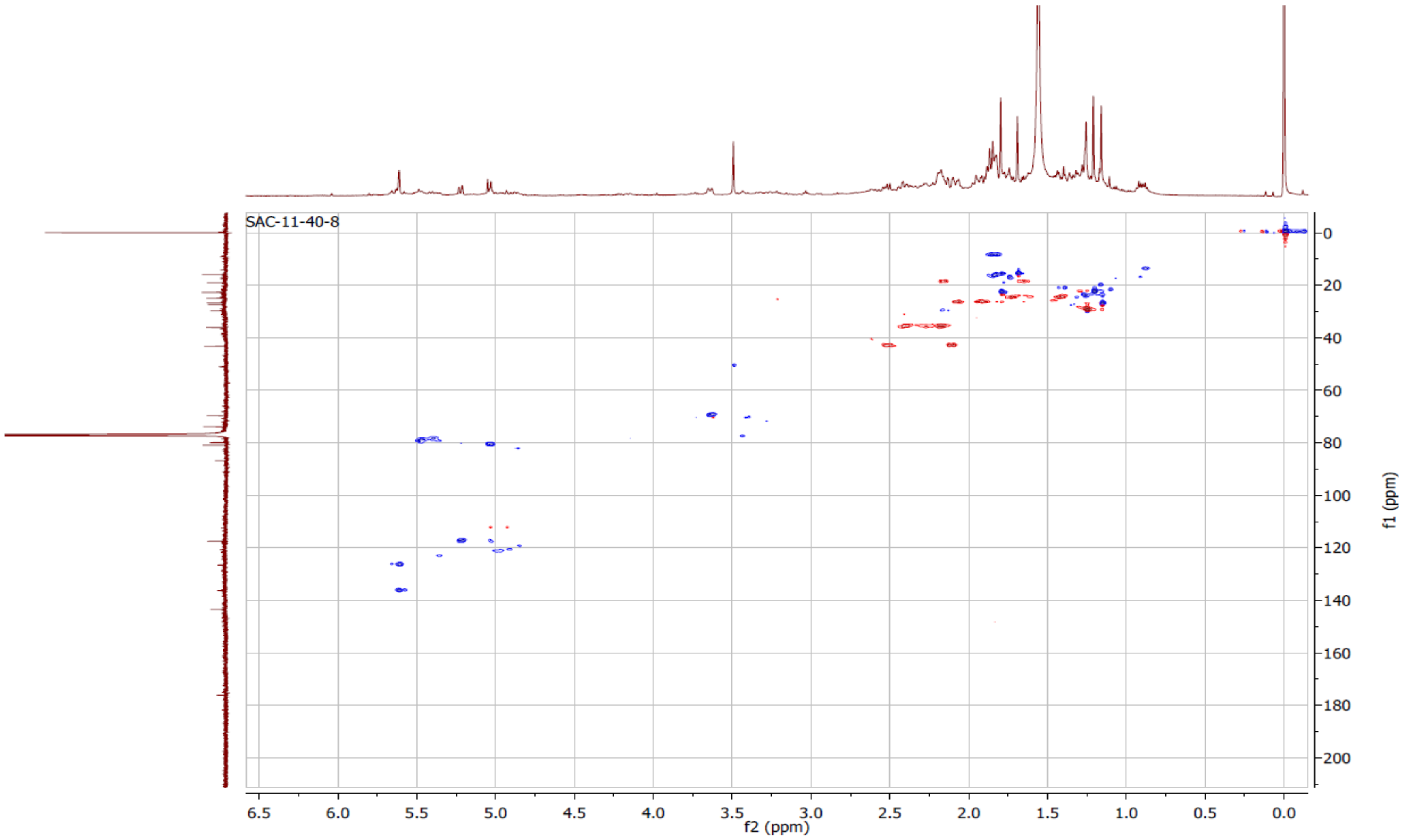

Figure S4. HSQC of **1.**

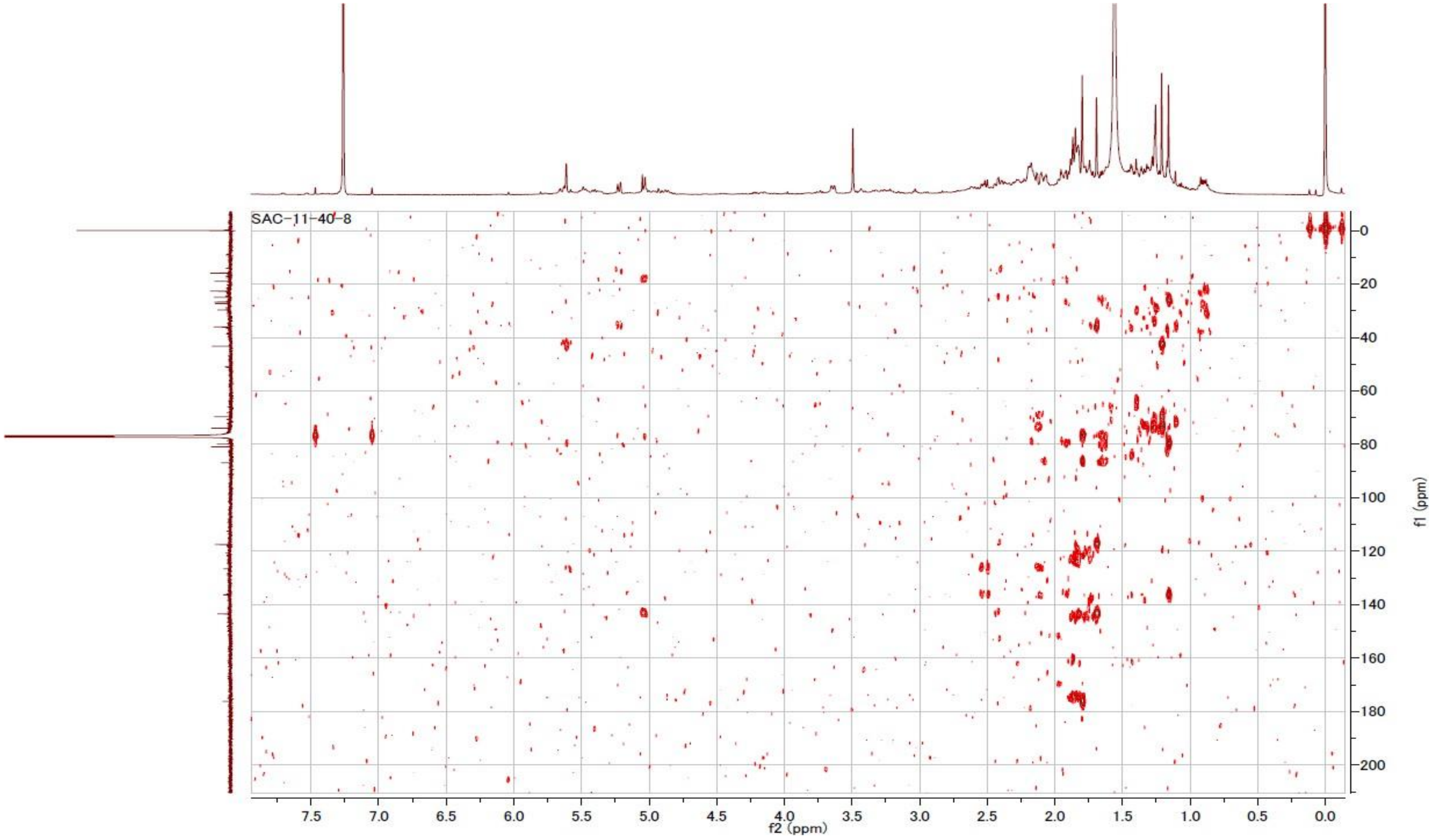

Figure S5. HMBC of **1.**

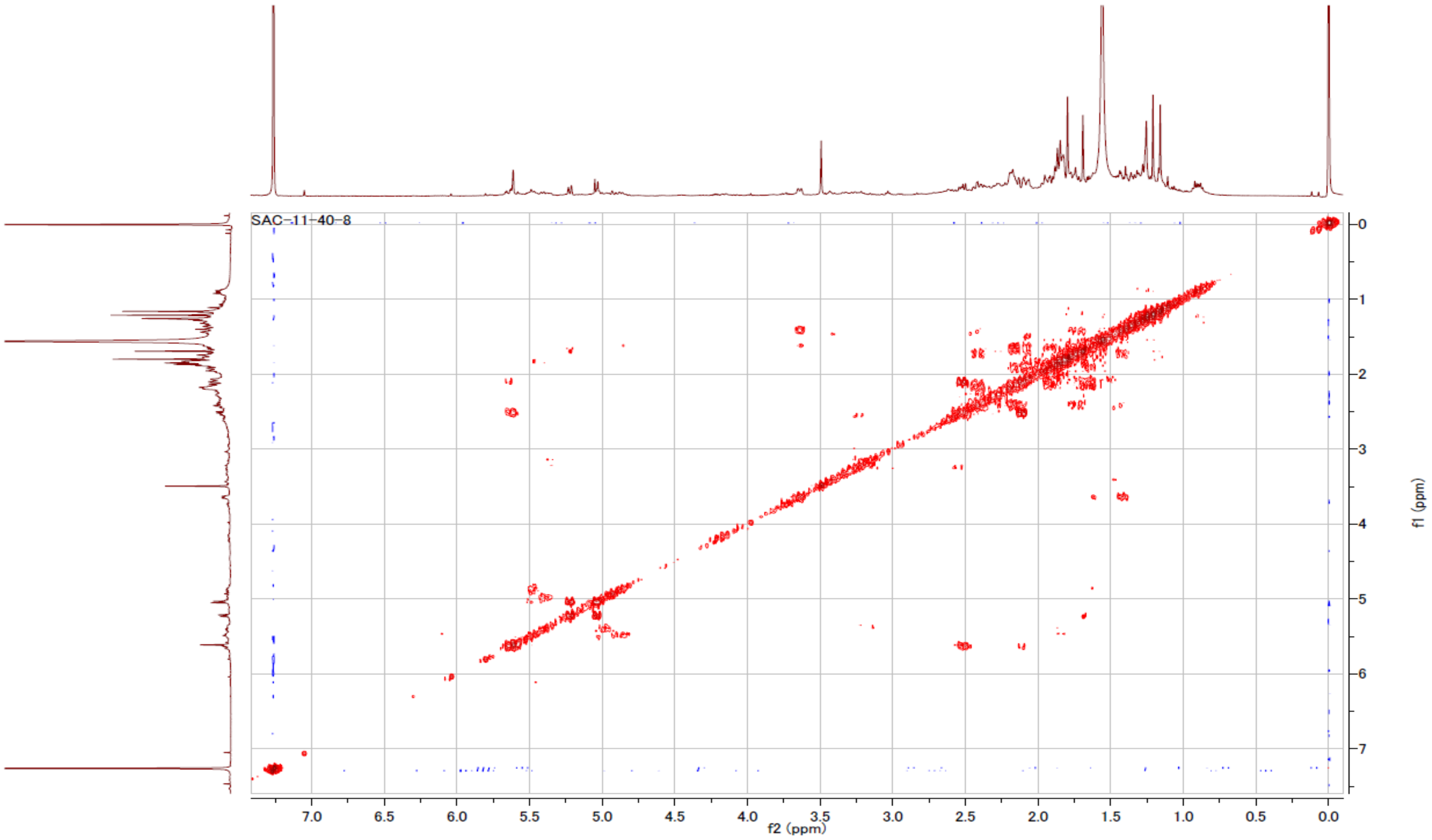

Figure S6. ^{1}H-^{1}H COSY of **1.**

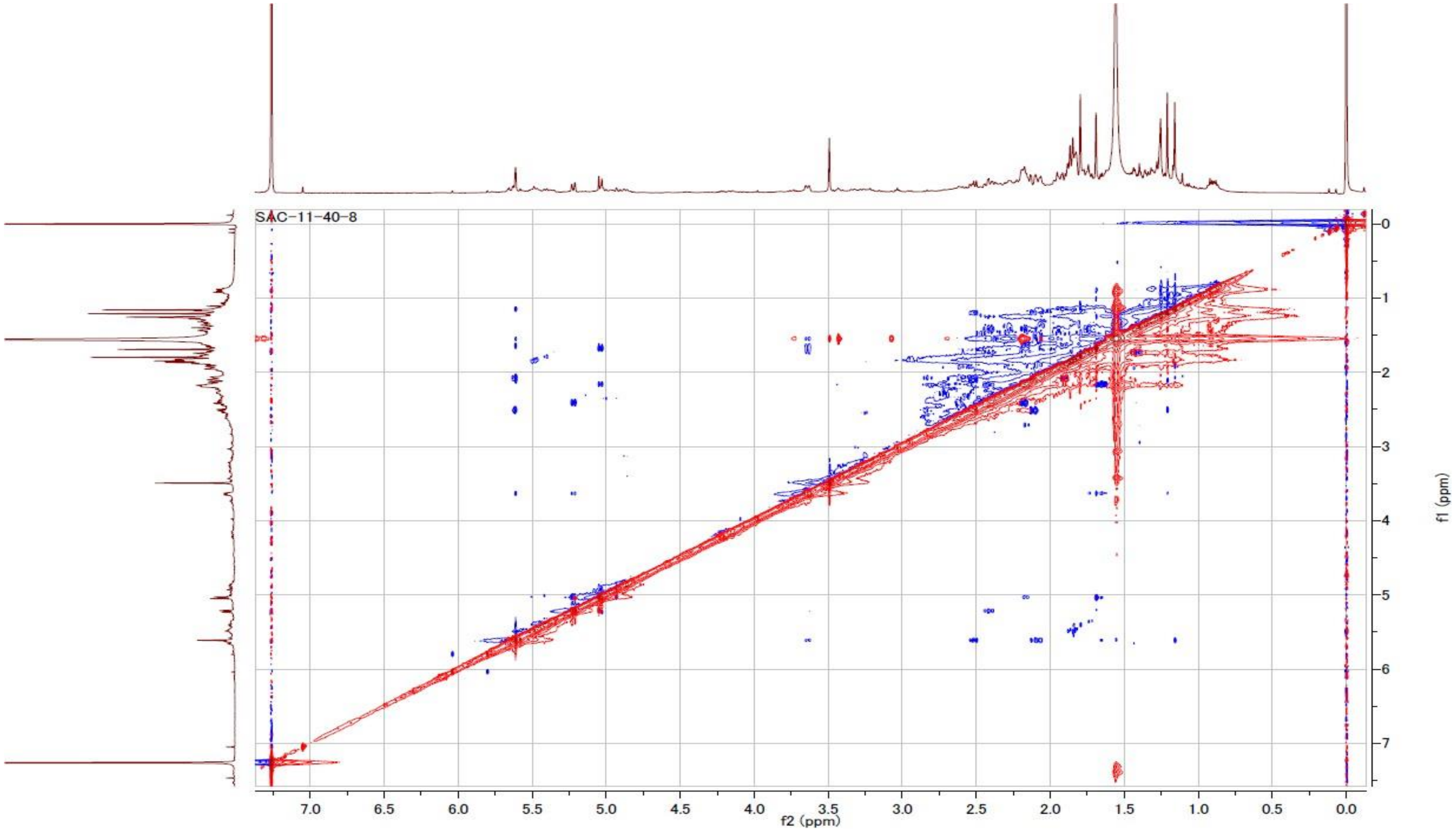

Figure S7. NOESY of **1.**

Data: Umeyama-CIHR. 07-Aug-2019.004 Date: 07-Aug-2019 17:02
Instrument: MStation
Sample: SAS-11-40-8
Note: MStation
Inlet: Direct Ion Mode: CI+
RT: 1.04 Scan#: 27
Elements: C 150/0, H 250/0, 50/0
Mass Tolerance: 5mmu
Unsaturation (U.S.): 0.0 – 15.0

	Observed *M/Z*	Int. %	Err. [ppm / mmu]	U.S.	Composition
1	400.2094	16.23	- 0.8 / -0.3	1.5	C20 H32 O8

Figure S8. HRCI/mas of **1.**

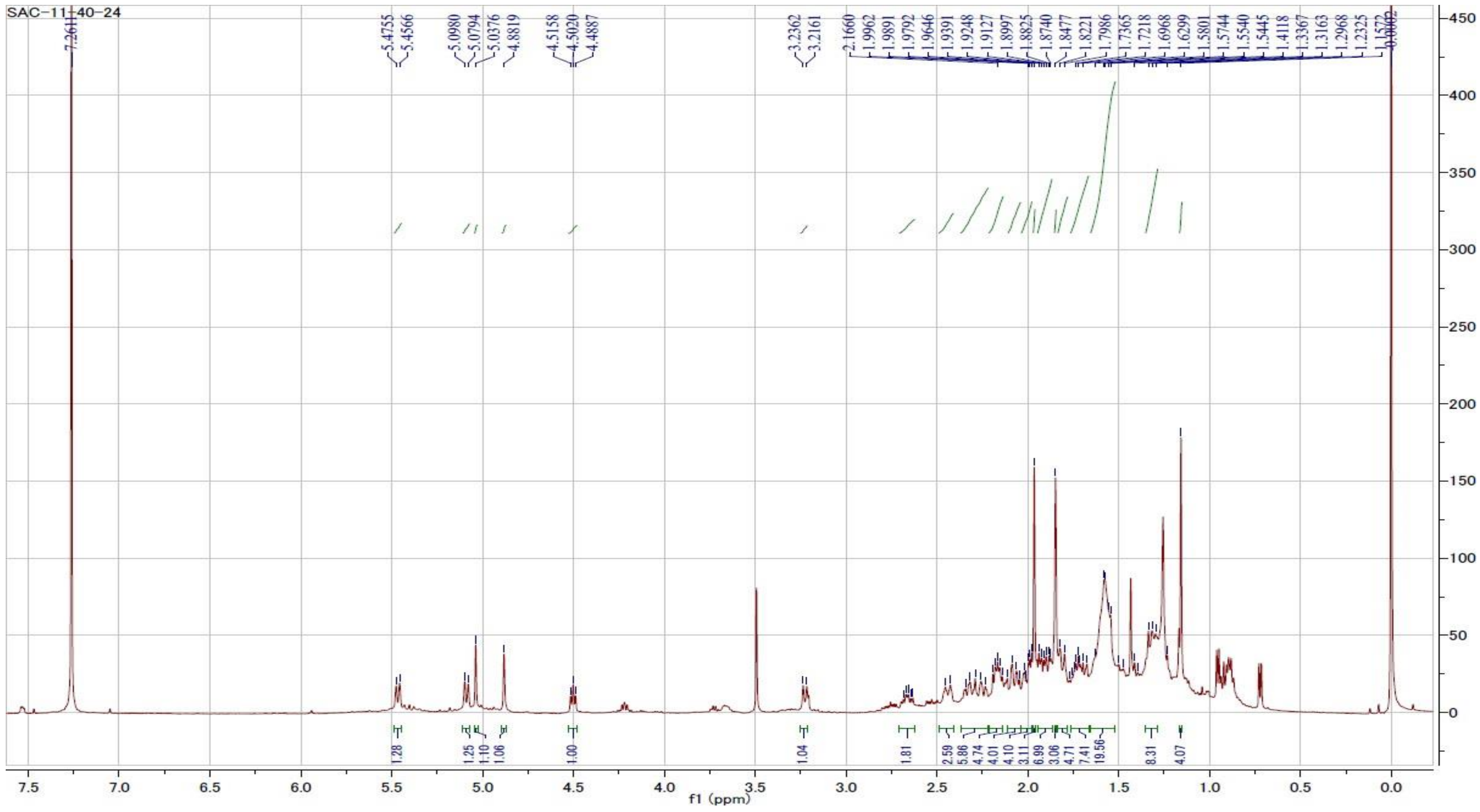

Figure S9. H NMR (CDCl3, 500 MHz) of **2.**

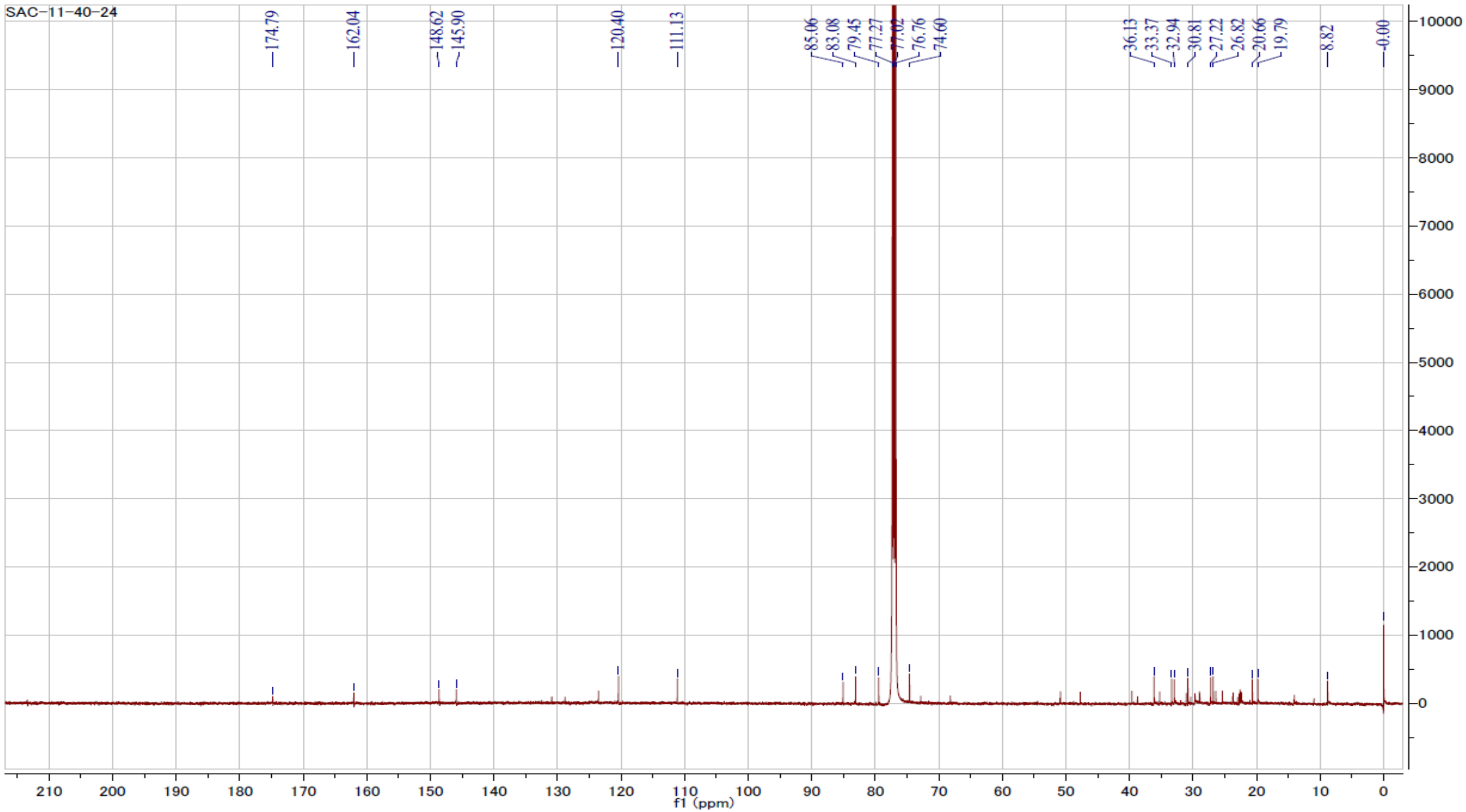

Figure S10. C NMR (CDCl3, 125 MHz) of **2.**

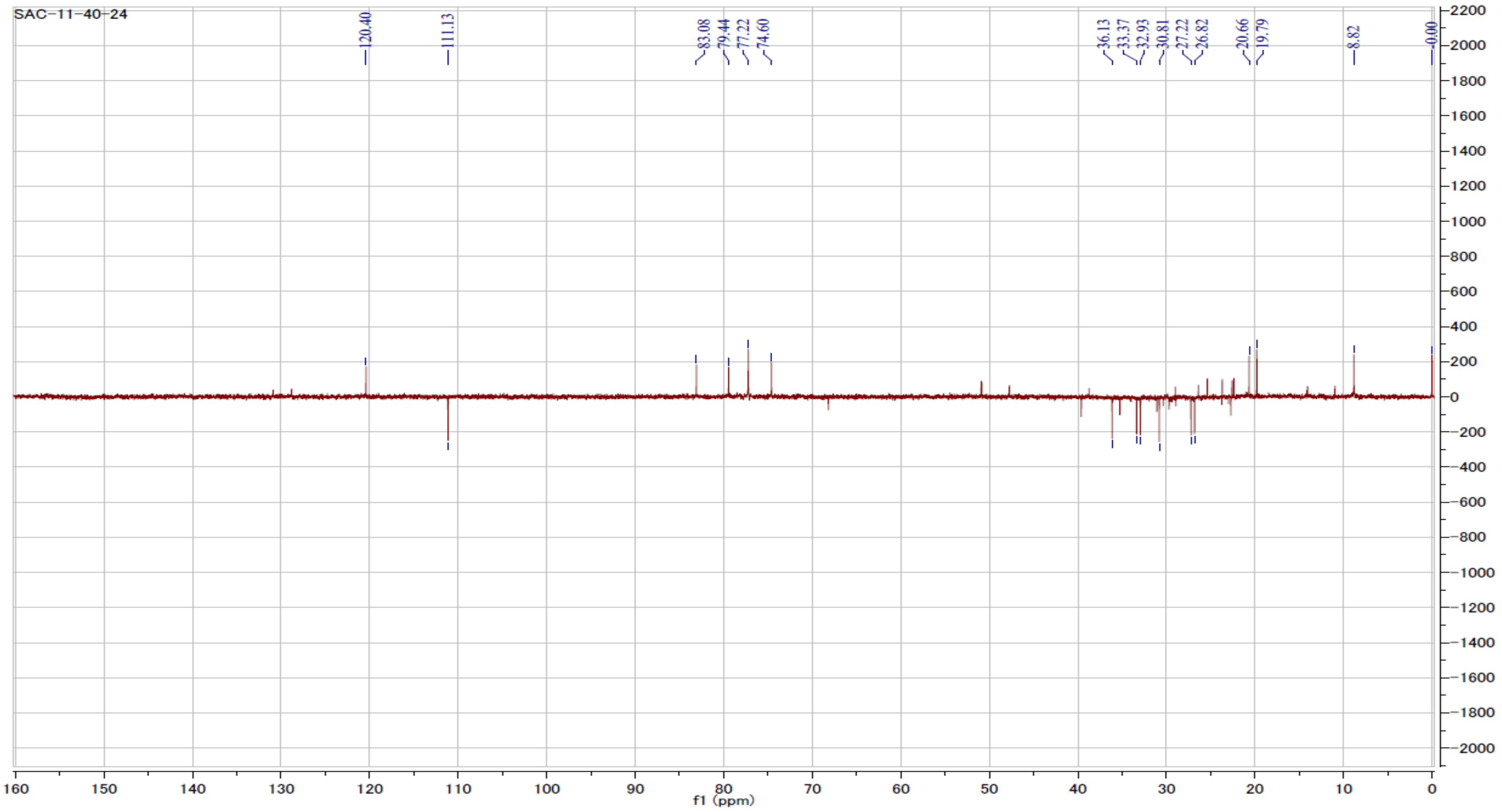

Figure S11. DEPT of **2.**

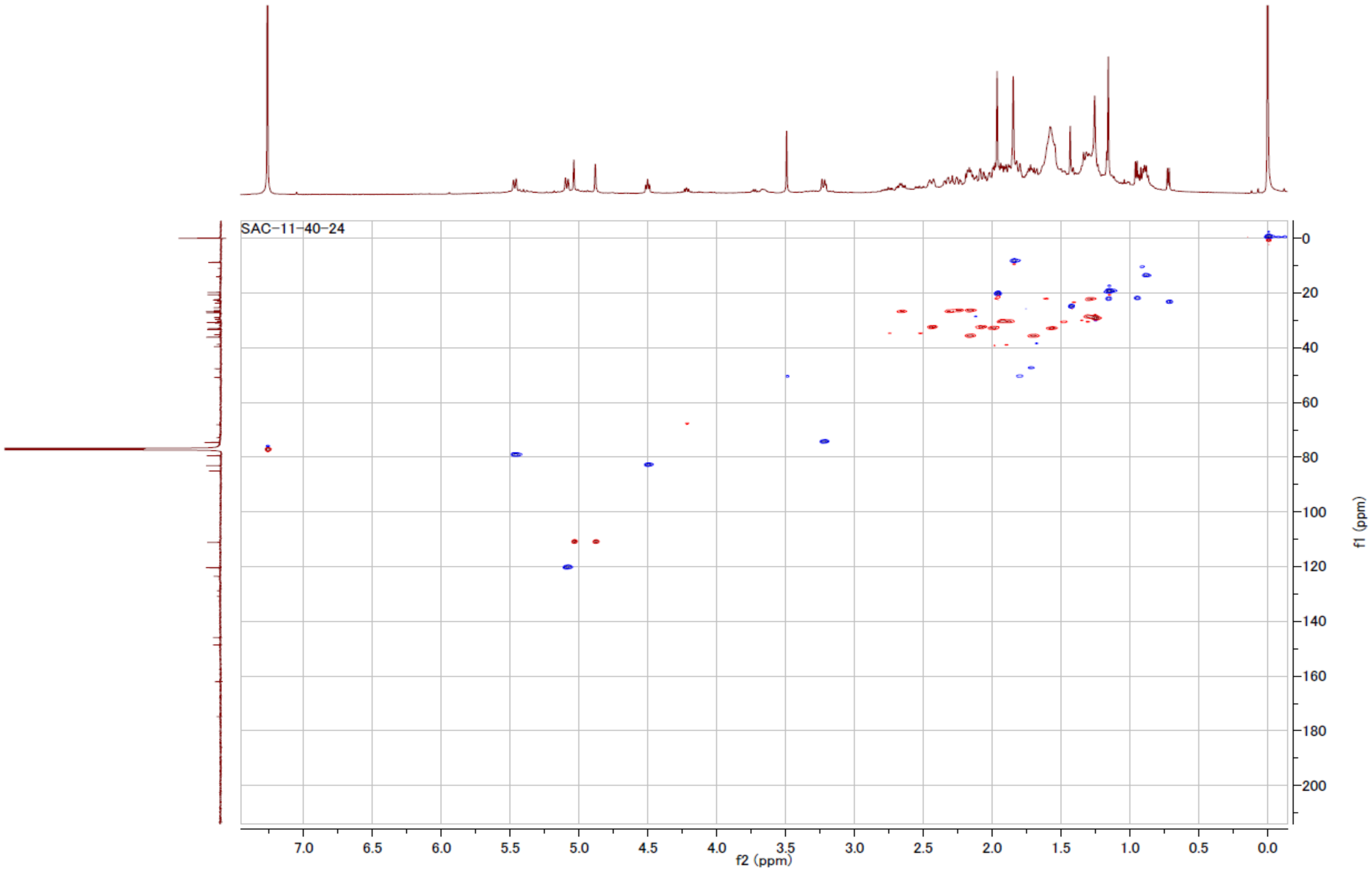

Figure S12. HSQC of **2.**

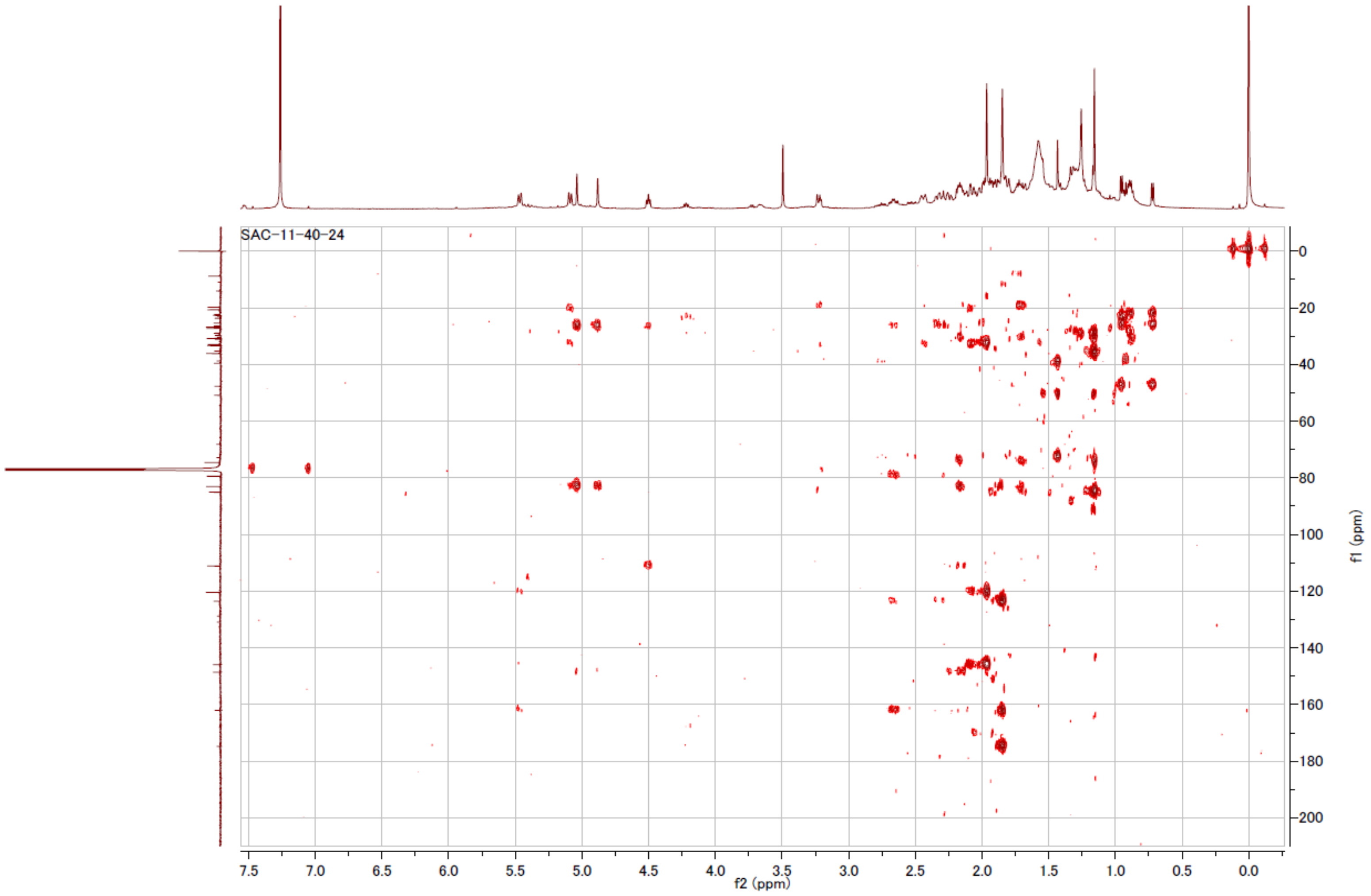

Figure S13. HMBC of **2.**

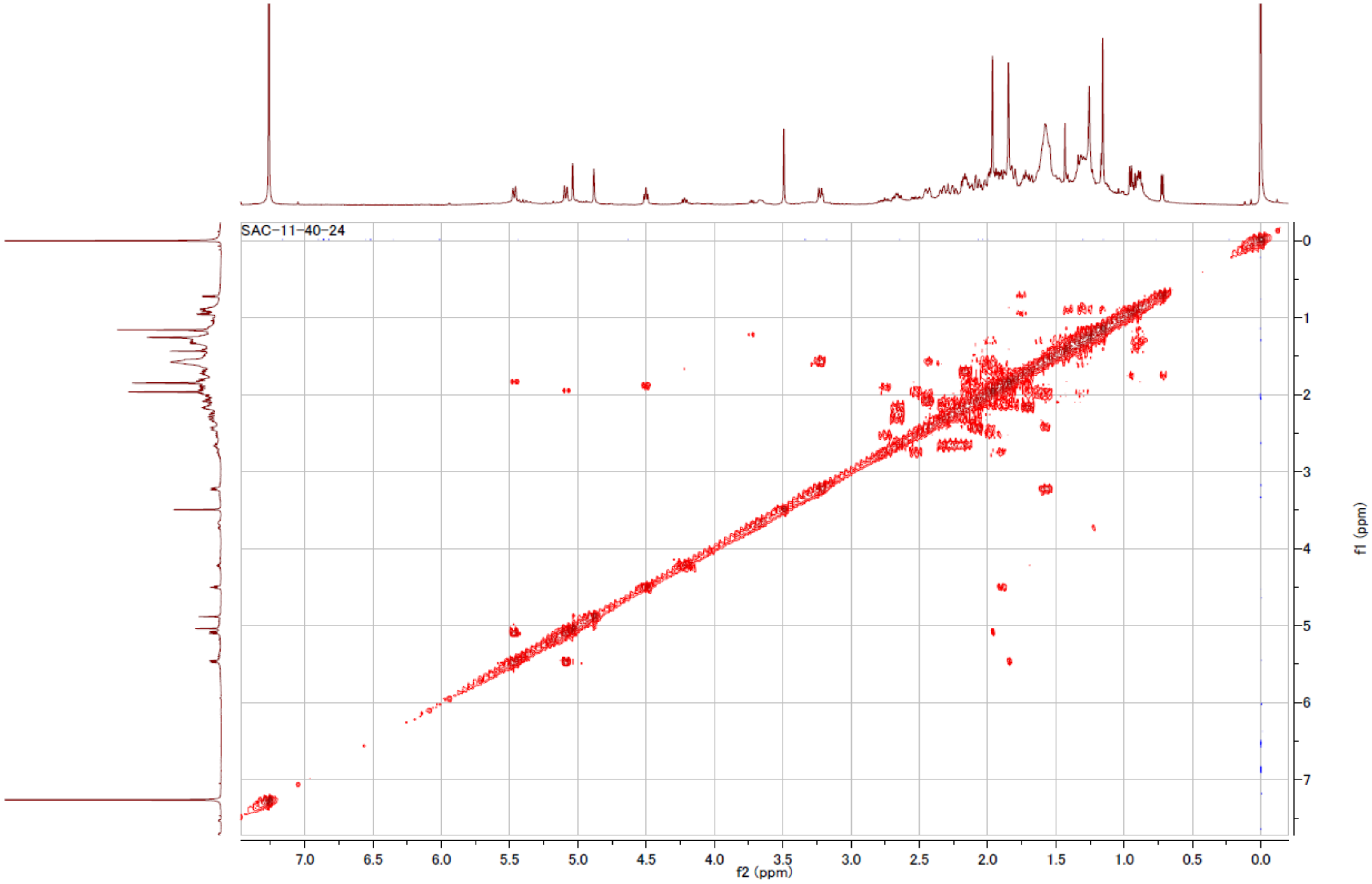

Figure S14. ¹H-¹H COSY of **2.**

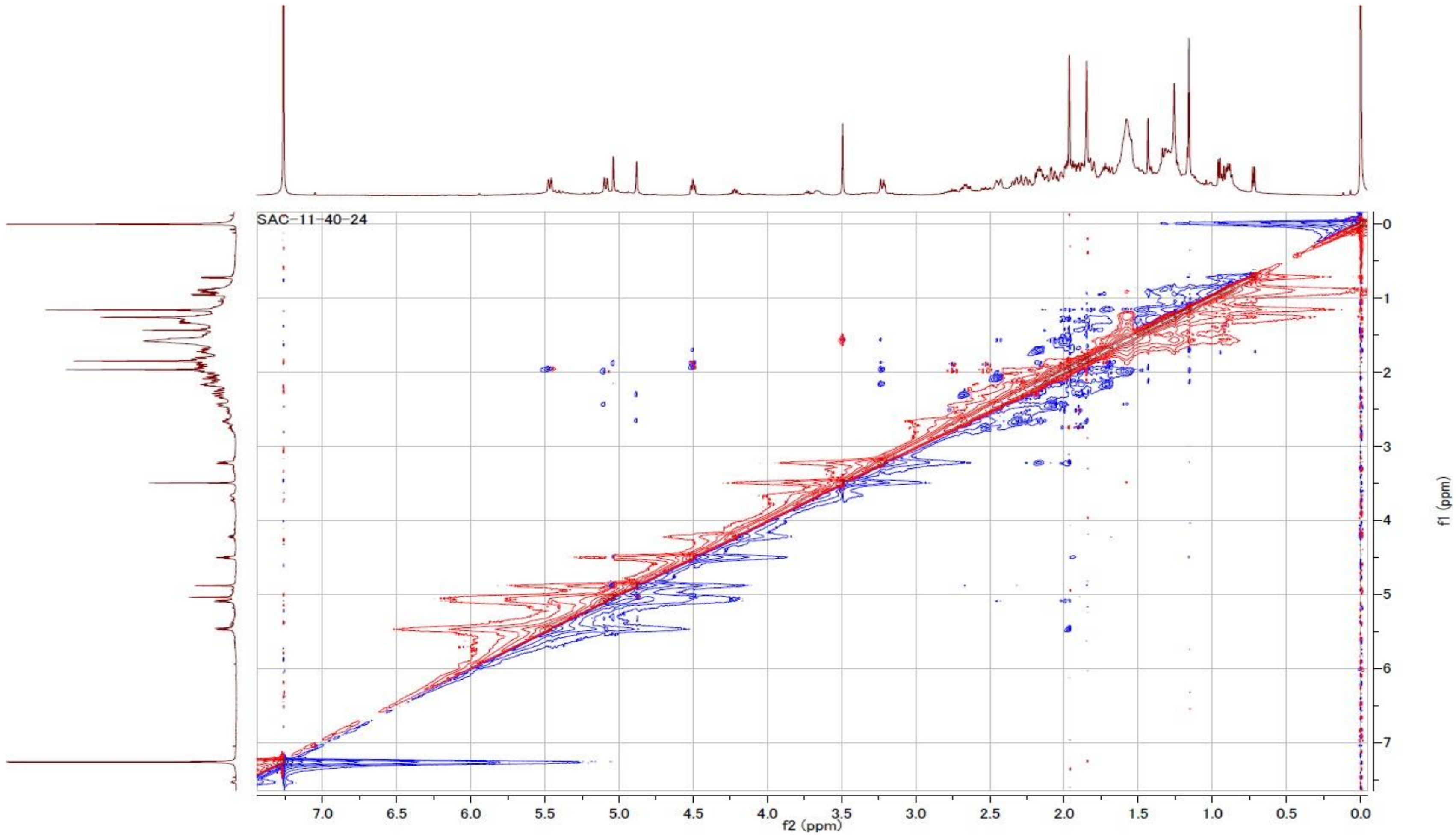

Figure S15. NOESY of **2.**

[Mass Spectrum]
Data : Umeyama-CI.25-Mar-2019.001 Date : 25-Mar-2019 16:17
Sample : SAC-11-40-24(CH4)
Note : MStation
Inlet : Direct Ion Mode : CI+
Spectrum Type : Normal Ion [MF-Linear]
RT : 1.75 min Scan# : 65
BP : m/z 332 Int. : 79.33 (831792)
Output m/z range : 35 to 500 Cut Level : 0.00 %

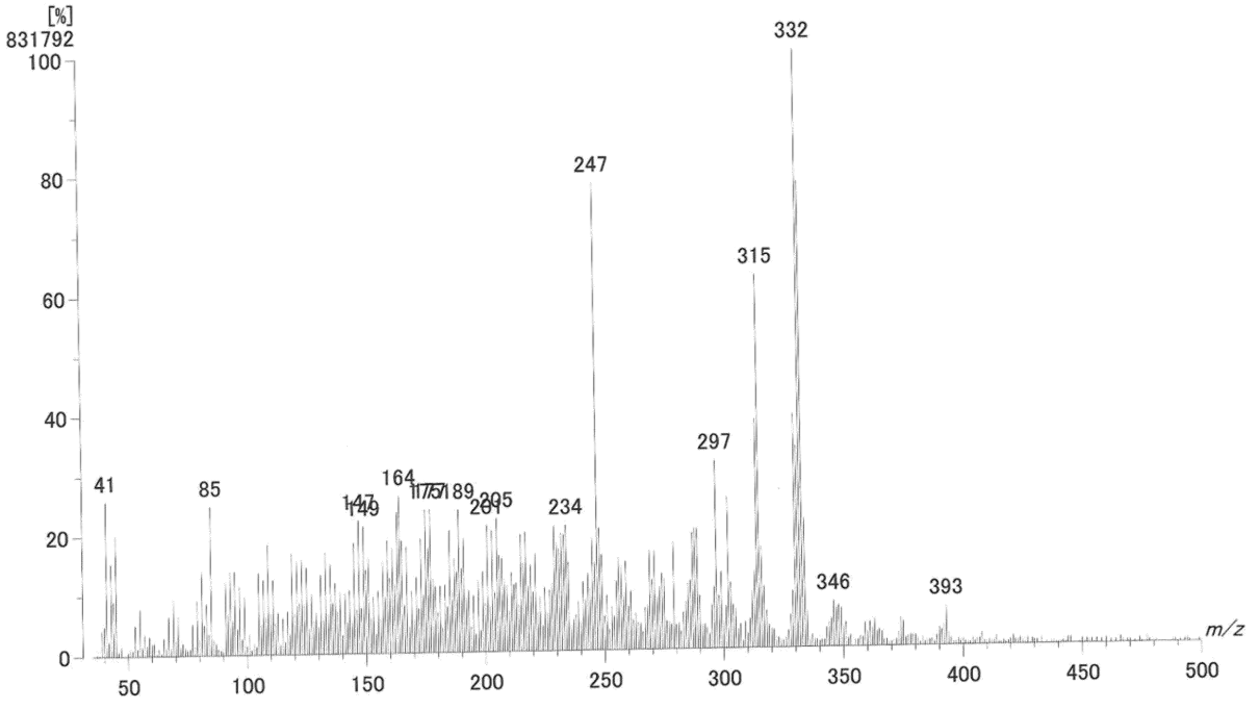

Figure S16. CI/mas of **2.**

Data: Umeyama-EIHR. 27-Mar-2019.003 Date: 27-Mar-2019 08:10
Instrument: MS700D

Sample: SAS-11-40-24

Note: MStation
Inlet: Direct Ion Mode: EI+
RT: 1.68 Scan#: 43
Elements: C 150/0, H 250/0, 50/0
Mass Tolerance: 5mmu
Unsaturation (U.S.): 0.0 - 20

	Observed *M/Z*	Int. %	Err. [ppm / mmu]	U.S.	Composition
1	332.1991	14.53	- 1.1 / -0.7	6.0	C20 H28 O4

Figure 17. HRCI/mas of **2.**

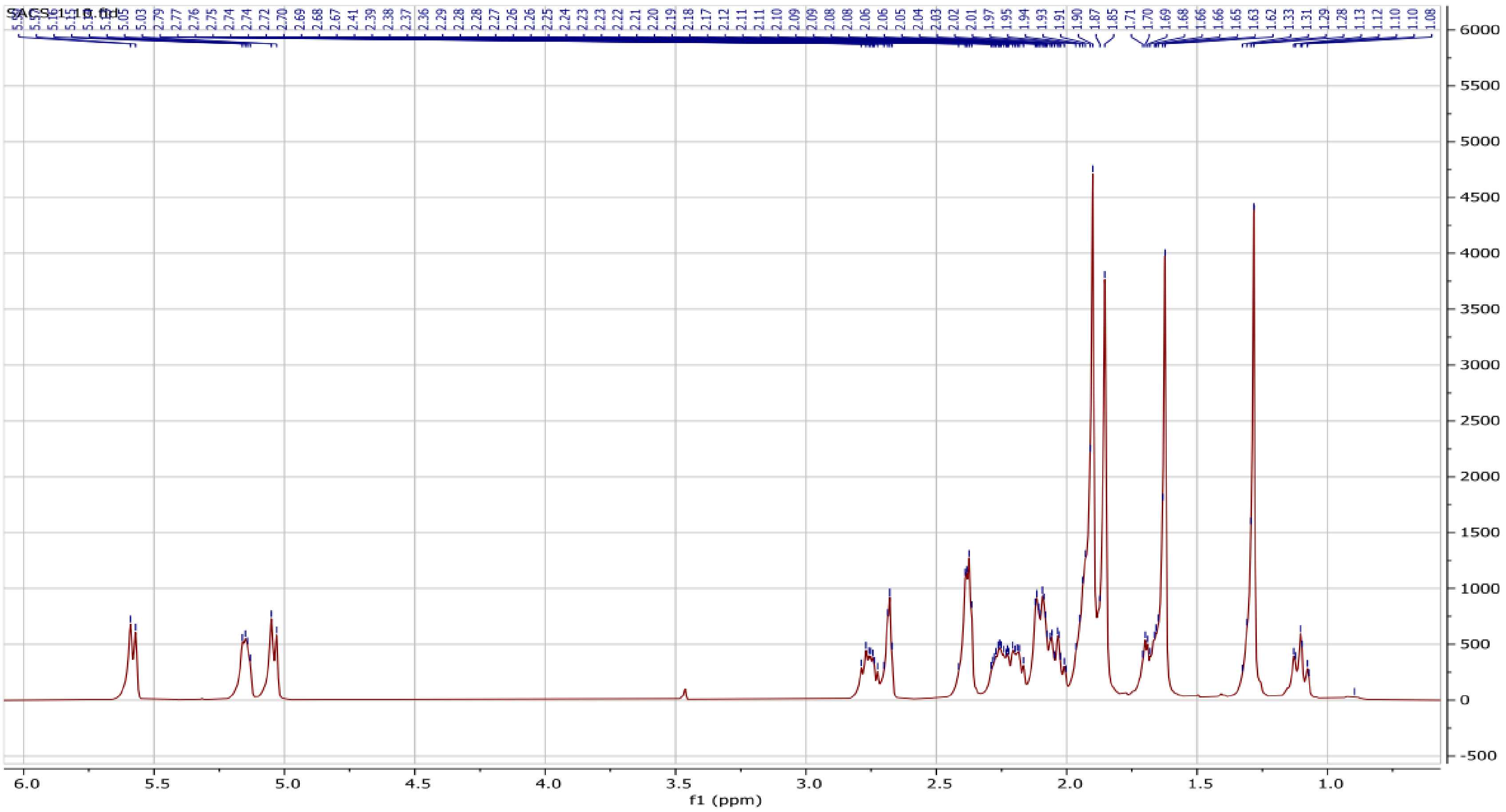

Figure S18. ^{1}H NMR($CDCl_3$, 500 MHz) of **3**.

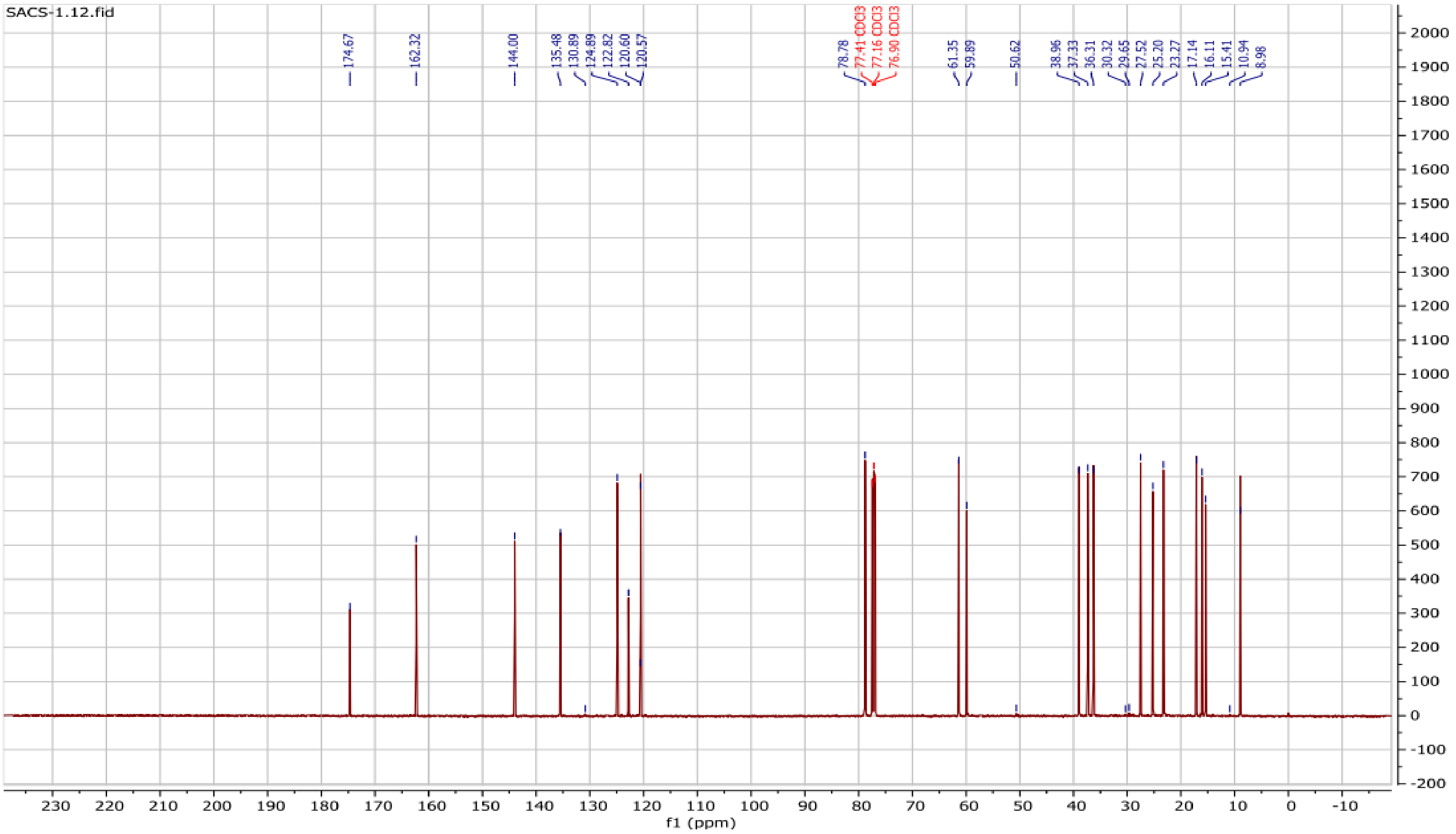

Figure S19. ^{13}C NMR (C_5D_5N, 125 MHz) of **3.**

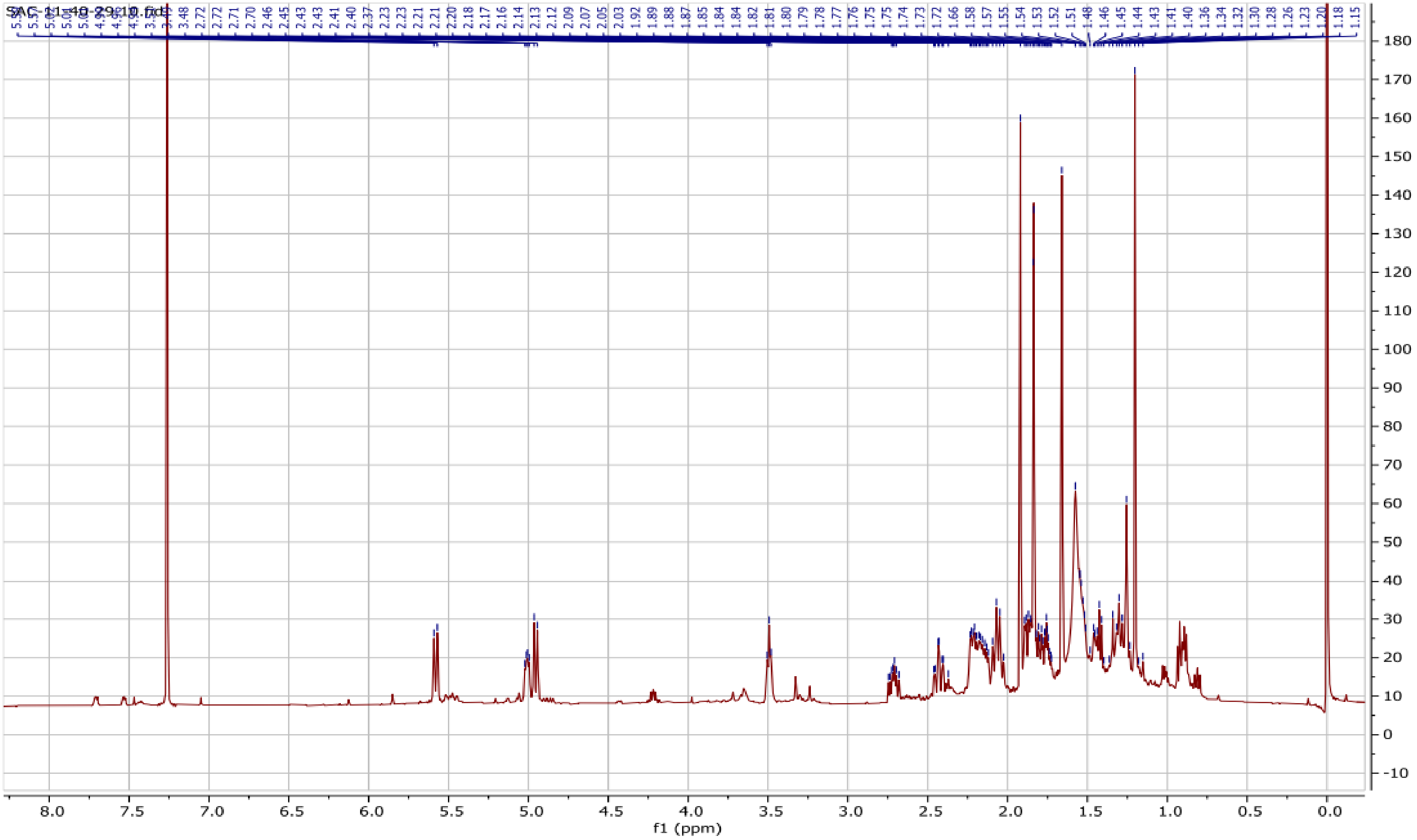

Figure S20. ^{1}H NMR ($CDCl_3$, 500 MHz) of **4.**

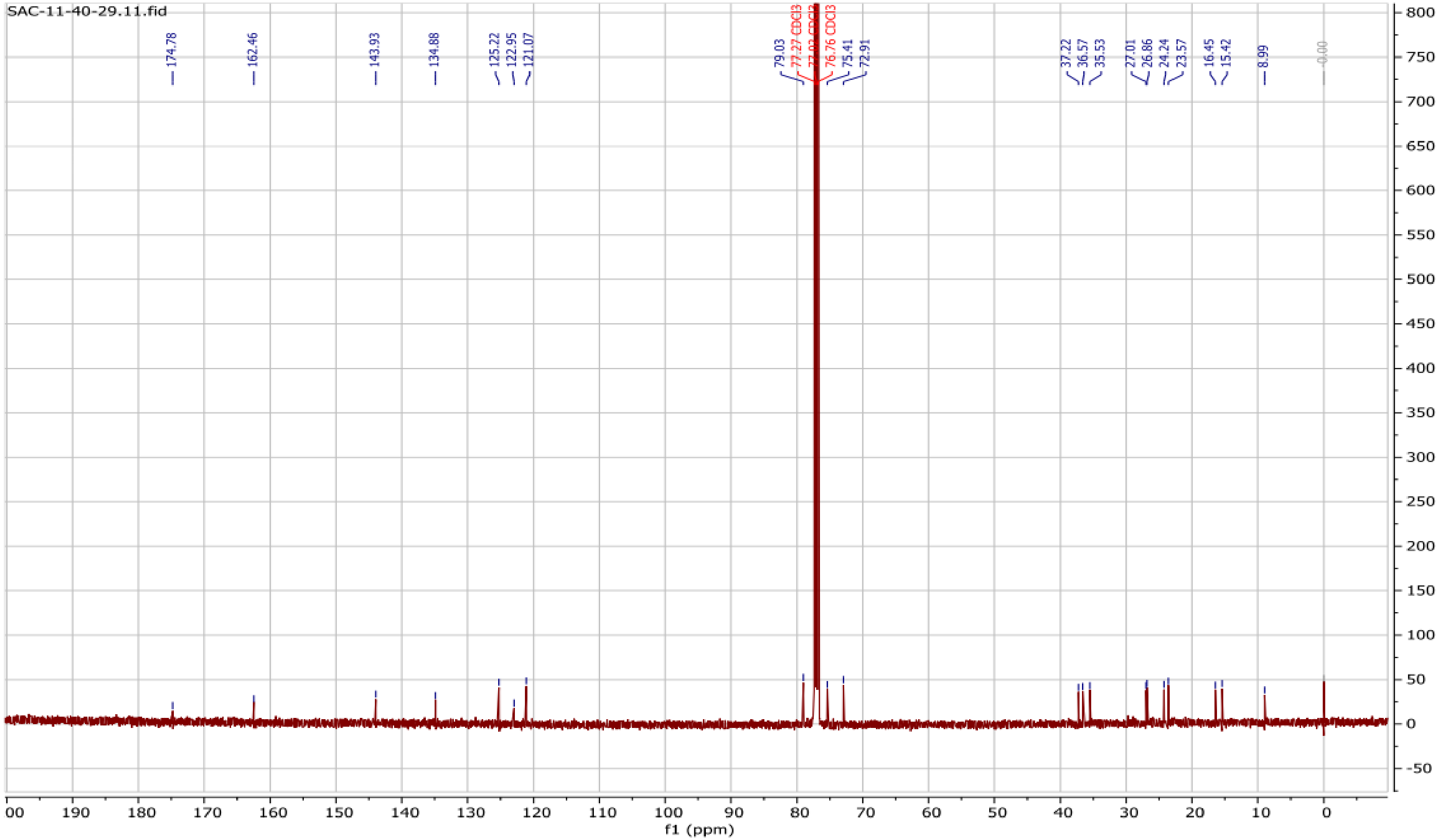

Figure S21. ^{13}C NMR (C_5D_5N, 125 MHz) of **4.**

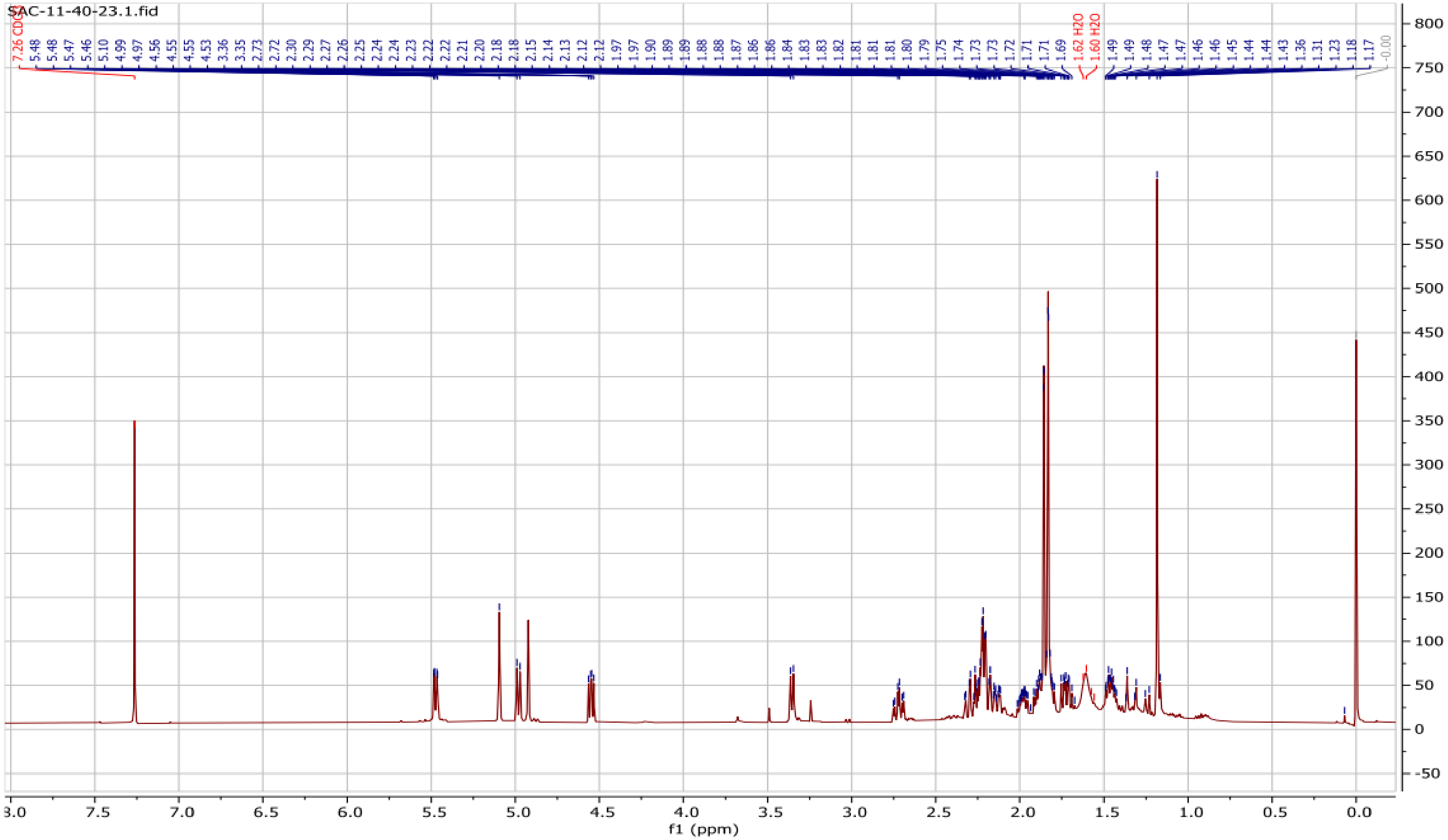

Figure S22. ^{1}H NMR ($CDCl_3$, 500 MHz) of **5.**

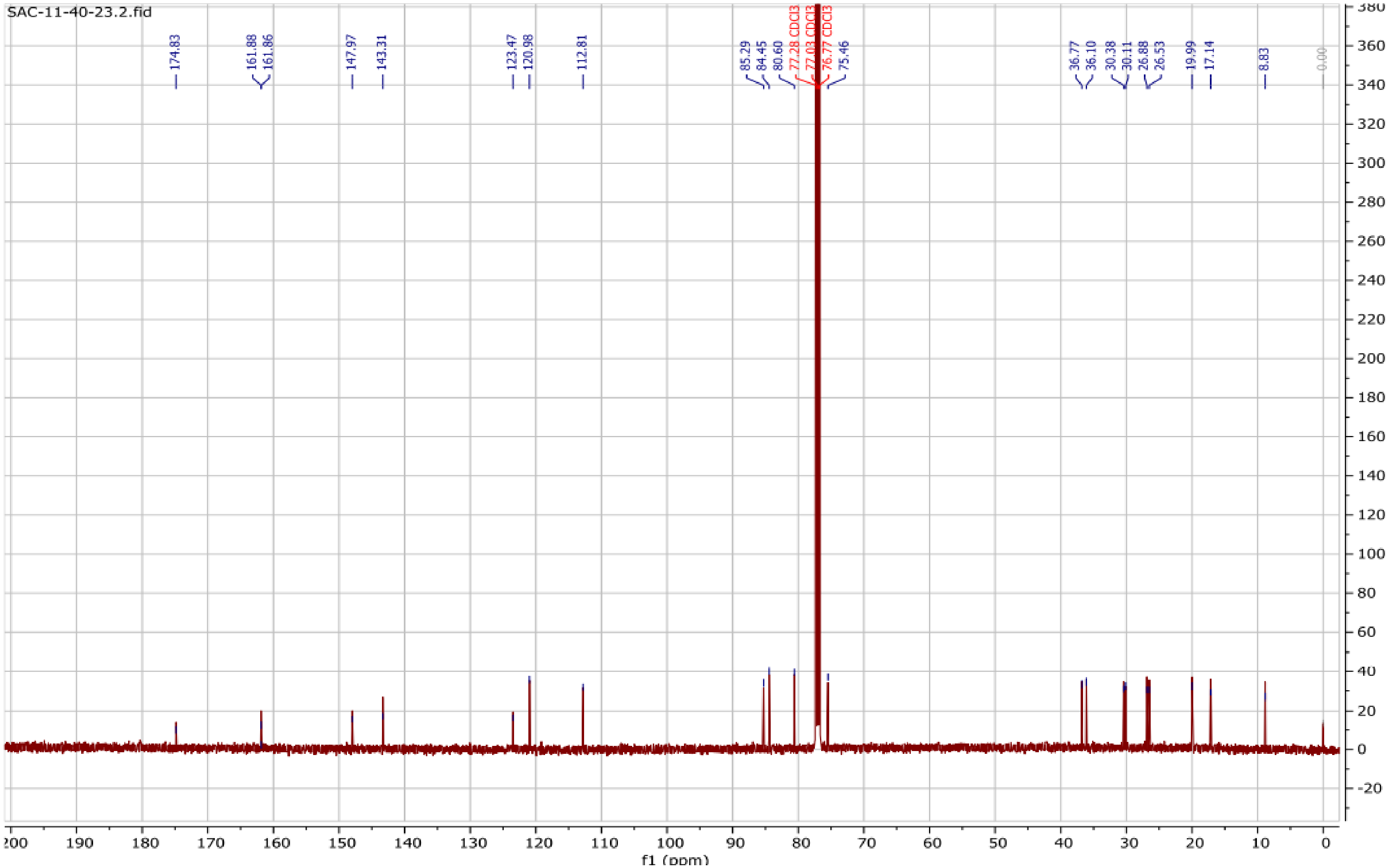

Figure S23. ^{13}C NMR (C_5D_5N, 125 MHz) of **5**.

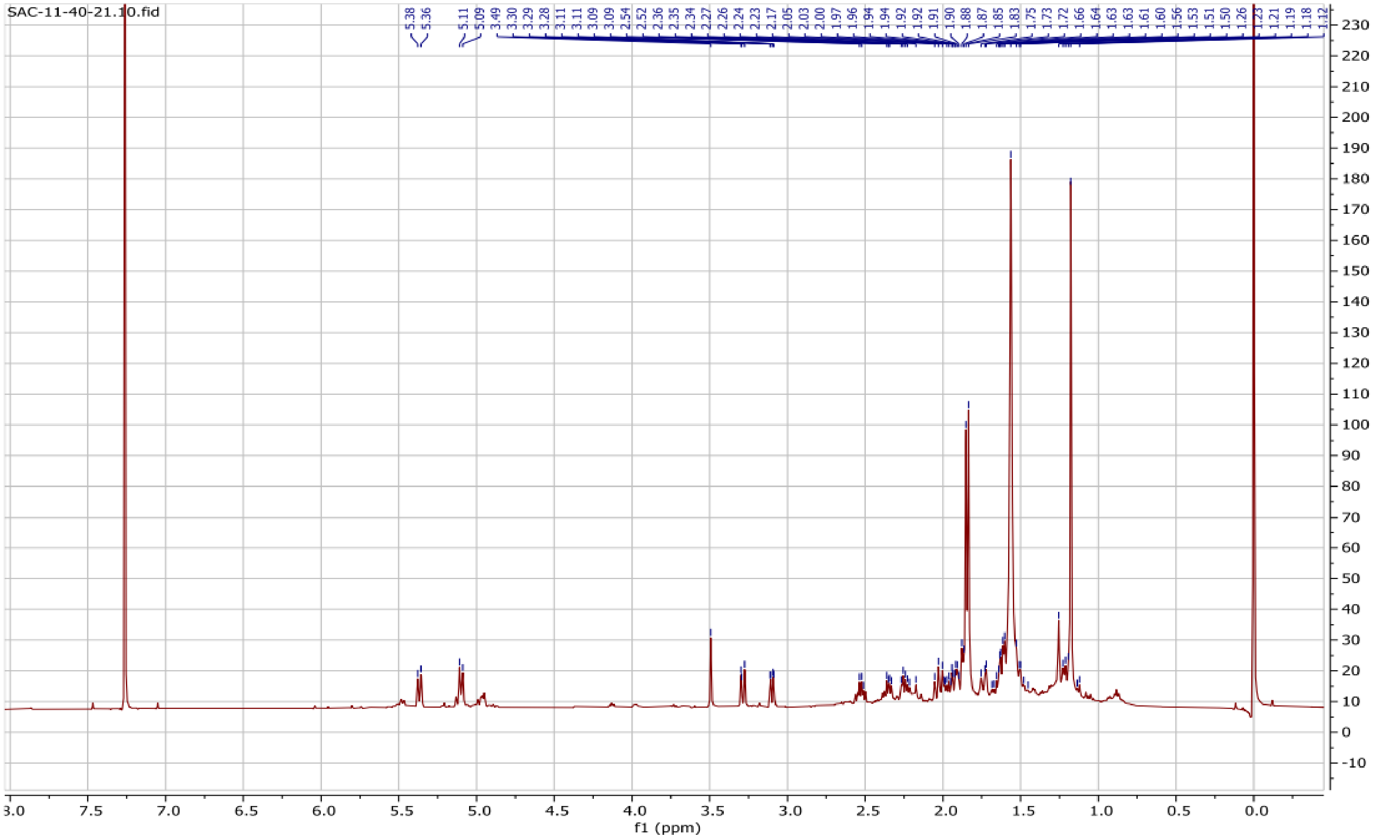

Figure S24. ^{1}H NMR ($CDCl_3$, 500 MHz) of **6**.

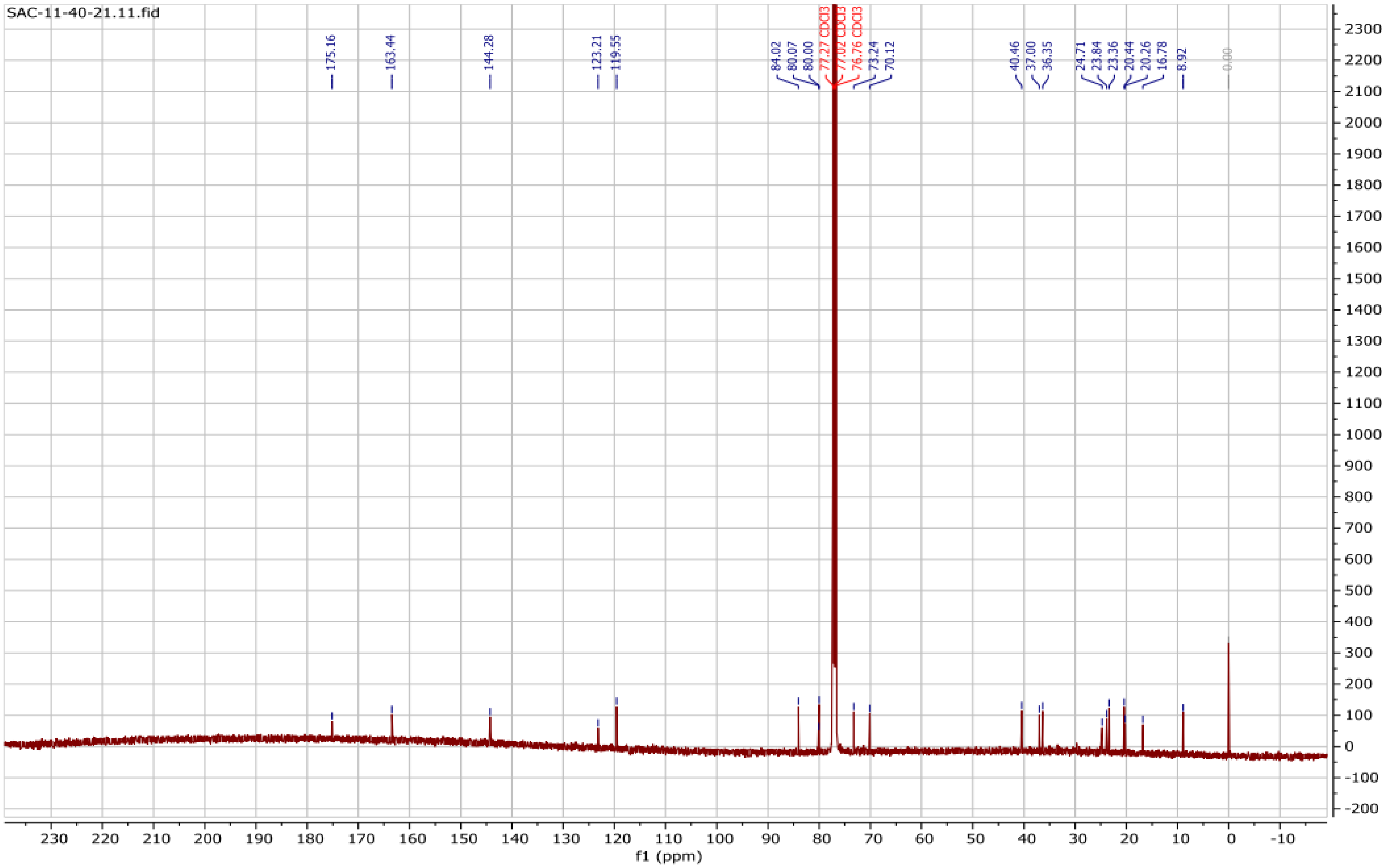

Figure S25. ^{13}C NMR (C_5D_5N, 125 MHz) of **6.**

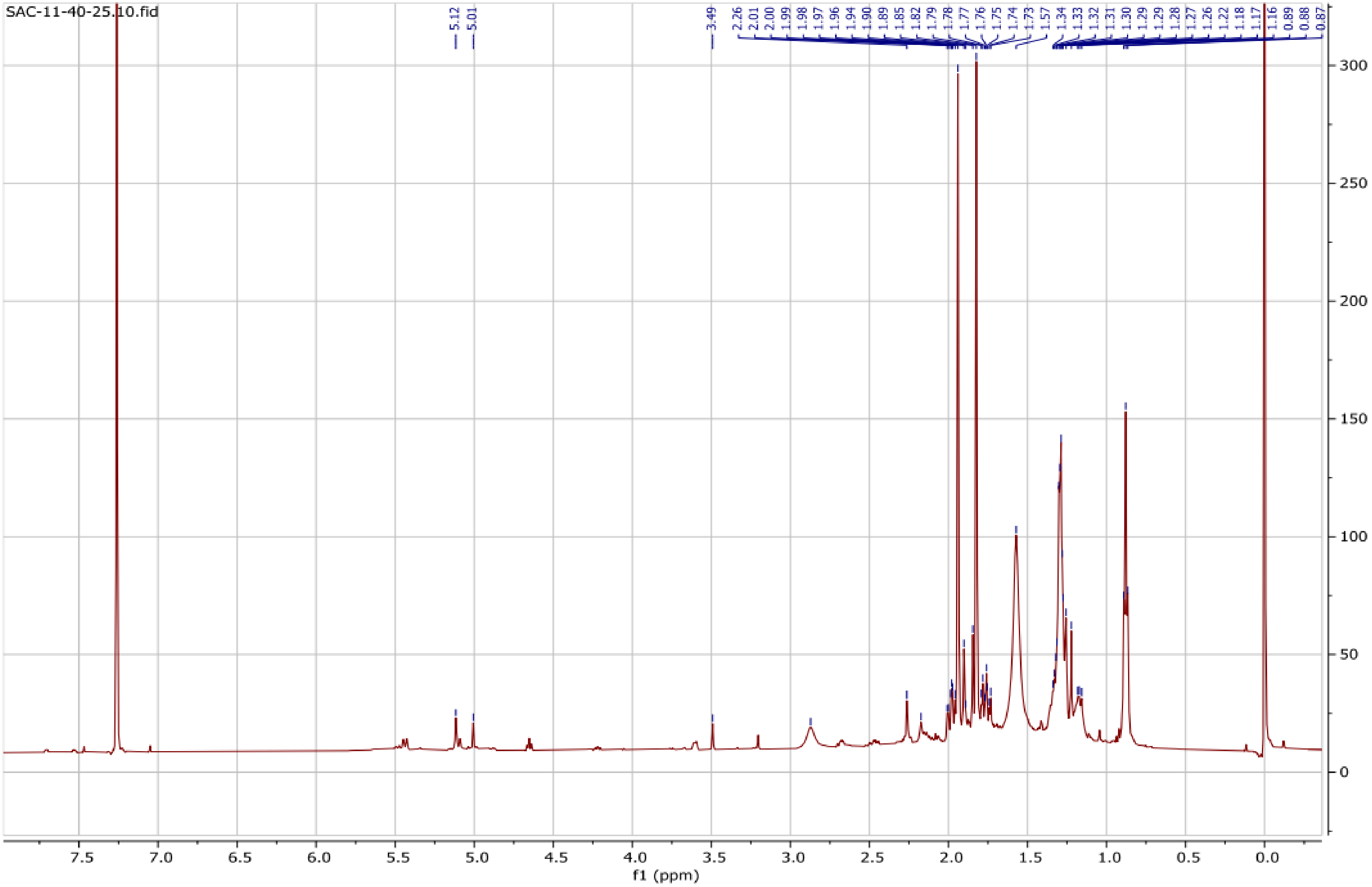

Figure S26. ^{1}H NMR ($CDCl_3$, 500 MHz) of **7.**

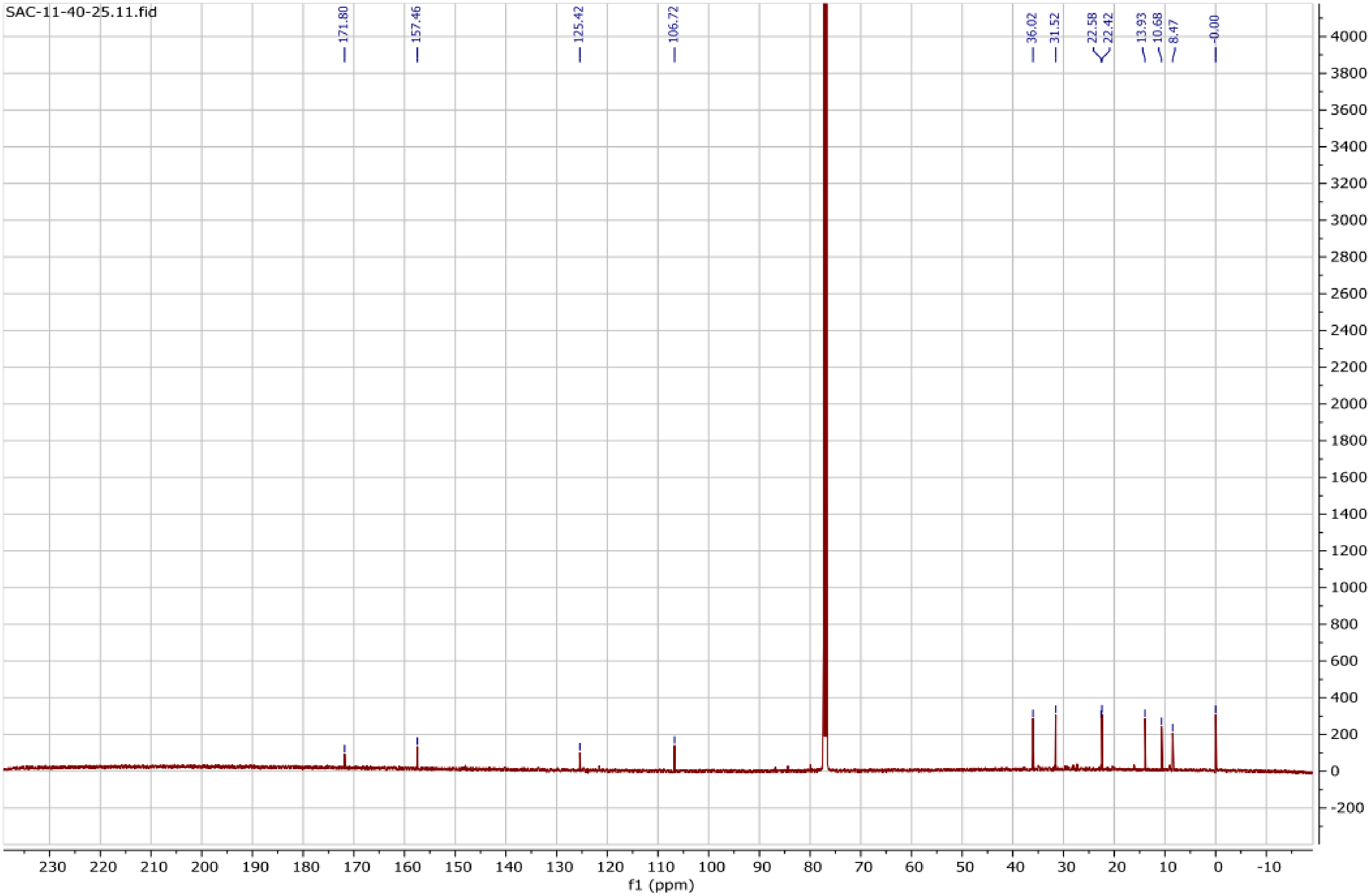

Figure S27. ^{13}C NMR (C_5D_5N, 125 MHz) of **7**.
